# Genetic Variants Associated with Port-Wine Stains

**DOI:** 10.1371/journal.pone.0133158

**Published:** 2015-07-20

**Authors:** Alice Frigerio, Karol Wright, Whitney Wooderchak-Donahue, Oon T. Tan, Rebecca Margraf, David A. Stevenson, J. Fredrik Grimmer, Pinar Bayrak-Toydemir

**Affiliations:** 1 Carolyn and Peter Lynch Center for Laser and Reconstructive Surgery, Division of Facial Plastic and Reconstructive Surgery, Department of Otology and Laryngology, Massachusetts Eye and Ear Infirmary, Harvard Medical School, Boston, MA, United States of America; 2 ARUP Institute for Clinical and Experimental Pathology, Salt Lake City, UT, United States of America; 3 Department of Pathology, University of Utah, Salt Lake City, UT, United States of America; 4 Division of Medical Genetics, Department of Pediatrics, Stanford University, Stanford, CA, United States of America; 5 Division of Otolaryngology, Department of Surgery, University of Utah, Salt Lake City, UT, United States of America; Pavillon Kirmisson, FRANCE

## Abstract

**Background:**

Port-wine stains (PWS) are capillary malformations, typically located in the dermis of the head and neck, affecting 0.3% of the population. Current theories suggest that port-wine stains are caused by somatic mutations that disrupt vascular development.

**Objectives:**

Understanding PWS genetic determinants could provide insight into new treatments.

**Methods:**

Our study used a custom next generation sequencing (NGS) panel and digital polymerase chain reaction to investigate genetic variants in 12 individuals with isolated port-wine stains. Importantly, affected and healthy skin tissue from the same individual were compared. A subtractive correction method was developed to eliminate background noise from NGS data. This allowed the detection of a very low level of mosaicism.

**Results:**

A novel somatic variant *GNAQ*, c.547C>G, p.Arg183Gly was found in one case with 4% allele frequency. The previously reported *GNAQ* c.548G>A, p.Arg183Gln was confirmed in 9 of 12 cases with an allele frequency ranging from 1.73 to 7.42%. Digital polymerase chain reaction confirmed novel variants detected by next generation sequencing. Two novel somatic variants were also found in RASA1, although neither was predicted to be deleterious.

**Conclusions:**

This is the second largest study on isolated, non-syndromic PWS. Our data suggest that *GNAQ* is the main genetic determinant in this condition. Moreover, isolated port-wine stains are distinct from capillary malformations seen in *RASA1* disorders, which will be helpful in clinical evaluation.

## Introduction

Port-wine stains (PWS; OMIM 163000) are congenital cutaneous lesions that are located in the head and neck region in approximately 75% of cases. By histology, PWS are characterized by dilated capillary-like vessels with normal endothelial cells and decreased neuronal markers[1].Prevalence is relatively high occurring in 3 of 1,000 individuals [2]. Although most cases are sporadic, familial PWS have also been reported [3].

The pathogenic mechanism of PWS is unknown. One theory suggests that PWS birthmarks are caused by somatic mutations disrupting early vascular development [4]. Recently, Shirley *et al*. identified a somatic mutation in *GNAQ* (c.548G>A, p.Arg183Gln) in 12 of 13 PWS samples from individuals with isolated PWS [5]. *GNAQ* encodes for an alpha subunit in the Gq class, which mediates signals between the G protein coupled membrane receptor and downstream effectors to evoke pleiotropic effects in response to extracellular signals [5]. Although the role of *GNAQ* is important for pathogenesis, not all individuals with PWS have a *GNAQ* mutation, suggesting other genetic etiologies.

Capillary malformation-arteriovenous malformation syndrome (CM-AVM; OMIM 608354) is a familial syndrome caused by mutations in *RASA1* [6]. *RASA1* encodes the Ras p21 protein activator 1, which is involved in regulating cell differentiation and proliferation, likely during angiogenesis [6]. In addition to capillary malformations, CM-AVM patients can also have fast-flow arteriovenous malformations (AVM) or fistulas (AVF), and Parkes-Weber syndrome (PKWS; OMIM 608355) [7,8]. Recently, somatic second-hit *RASA1* mutations were identified in these dermal lesions [9]. One aim in this study was to investigate the presence of *RASA1* mutations in PWS.

We postulated that the development of PWS is multifactorial and individuals may benefit from personalized plans based on genetic susceptibilities. Identifying the genetic determinants of PWS may provide insight into new treatments [8]. Our study was designed to identify additional causative genetic variants while also investigating the frequency of the previously identified *GNAQ* c.548G>A variant in a cohort of individuals with isolated PWS. Two genes (*GNAQ* and *RASA1*) were selected based on published data. Genes were interrogated using a custom next generation sequencing (NGS) panel, which allows assessment of >1% mosaicism.

## Materials and Methods

### Samples

The Declaration of Helsinki protocols were followed. Written informed consent was obtained for all participants. Institutional review board approval was obtained by Massachusetts Eye and Ear Infirmary (MEEI) Human Studies Committee (IRB ID #391205).

Participants were recruited from adult PWS patients who entered the Carolyn and Peter Lynch Laser Center at MEEI for treatment of their condition.

Twelve participants (11 females, 1 male) ages 18–62 years were enrolled. All PWS were isolated, i.e. not associated with other vascular anomalies. PWS were phenotyped by means of 3dMD photogrammetric software (3dMD; Atlanta, GA) according to a method previously described [10]; location and side of each lesion are detailed in Table 1.

**Table 1:** Patient demographics and PWS location.

Case	Sex	Age (years)	PWS Location	Side
**1**	M	41	V2 dermatome	L
**2**	F	39	upper body	L
**3**	F	72	wrist	R
**4**	F	29	V1 dermatome	L
**5**	F	18	V3 dermatome	R,L
			C2 dermatome	R
**6**	F	33	upper body	R,L
**7**	F	62	V2 dermatome	L
			V3 dermatome	R,L
**8**	F	28	half body	R,L
**9**	F	26	shin	R
**10**	F	25	V1 dermatome	L
**11**	F	51	forearm	L
**12**	F	45	V2 dermatome	R

Abbreviations: V1, frontal trigeminal branch; V2, maxillary trigeminal branch; V3, mandibular trigeminal branch; R, right; L: left

Two 3 mm punch skin samples were collected from each participant. One intra-lesional biopsy was performed on untreated PWS skin; a second biopsy was performed on apparently normal skin of the contralateral, corresponding body region. Samples were flash frozen, stored at -80°C and shipped overnight on dry ice to ARUP Laboratories for DNA extraction and analysis. One healthy female peripheral blood sample was used as a negative control.

### Sample processing

DNA was extracted from 12 affected tissue samples and 12 unaffected tissue samples using the DNeasy Blood and Tissue Kit (Qiagen, Germantown, MD) with a three day proteinase K incubation at 56°C. DNA concentrations were verified using a NanoDrop 8000 (Thermo Scientific, Wilmington, DE).

### NGS Custom Capture

Custom capture RNA baits were designed to target the exons and exon/intron boundaries of the genes *GNAQ* and *RASA1* (~0.04Mb). DNA from affected and control tissue (1.4–3μg) were sheared to 180 bp fragments using a Covaris instrument (Covaris, Woburn, MA). Illumina adapters were added using the SureSelect XT kit reagents (Agilent Technologies, Santa Clara, CA). Adapter ligated libraries were then hybridized with the biotinylated RNA baits at 65°C for 24 hours. Hybridized DNA targets of interest were captured using streptavidin coated magnetic beads. Targeted DNA was washed, eluted, and then barcoded/indexed. DNA quality was assessed using a Bioanalyzer (Agilent Technologies, Santa Clara, CA).

### NGS Sequencing and Data Analysis

Genes were interrogated using an NGS custom capture method [11], which provides deep coverage and allows assessment of >1% mosaicism. Concentrations of the indexed sample libraries were verified using quantitative PCR (KAPA Biosystems, Willmington, MA) and pooled together at a 1:1 ratio. Samples were sequenced on the HiSeq2500 instrument (Illumina, San Diego, CA) using 2x100 paired-end reads. Sequences were aligned to the human genome reference (GRCh37) sequence, using Burrows-Wheeler Alignment (BWA 0.5.9) with default parameters. PCR duplicates were removed using Samtools, and base quality scores were recalibrated. Local realignment and variant calling were performed using the Genome Analysis Toolkit (GaTK v1.3). This method enables the detection of low level mosaic insertions and deletions.

Low frequency (<10%) single nucleotide variants were detected using a subtractive correction method described previously [12,13]. Read coverage was gathered from the bam file data for each bed file position, as well as for the three possible nucleotide changes from the reference at each position. Reads were filtered out if < 25 mapping quality or < 24 base. For each position, the variant read frequency of the unaffected tissue was subtracted from the variant read frequency for the affected tissue. Variants were kept for further analysis if the variant read frequency percentage was at least 1% higher in the affected tissue versus the unaffected tissue. Variants were analyzed using the Integrative Genomic Viewer.

### Digital PCR (dPCR)

Digital PCR was used to confirm the presence of somatic mutations (*GNAQ* p.Arg183Gln; *GNAQ* p.Arg183Gly) in the affected tissue samples. DNA from matched control tissues and one negative healthy control sample from peripheral blood were also evaluated. A custom TaqMan SNP Genotyping Assay was optimized for each variant using the QuantStudio 3D Digital PCR System (Life Technologies, Grand Island, NY). Results were analyzed using the QuantStudio 3D AnalysisSuite software relative quantification module.

## Results

**Fig 1:**
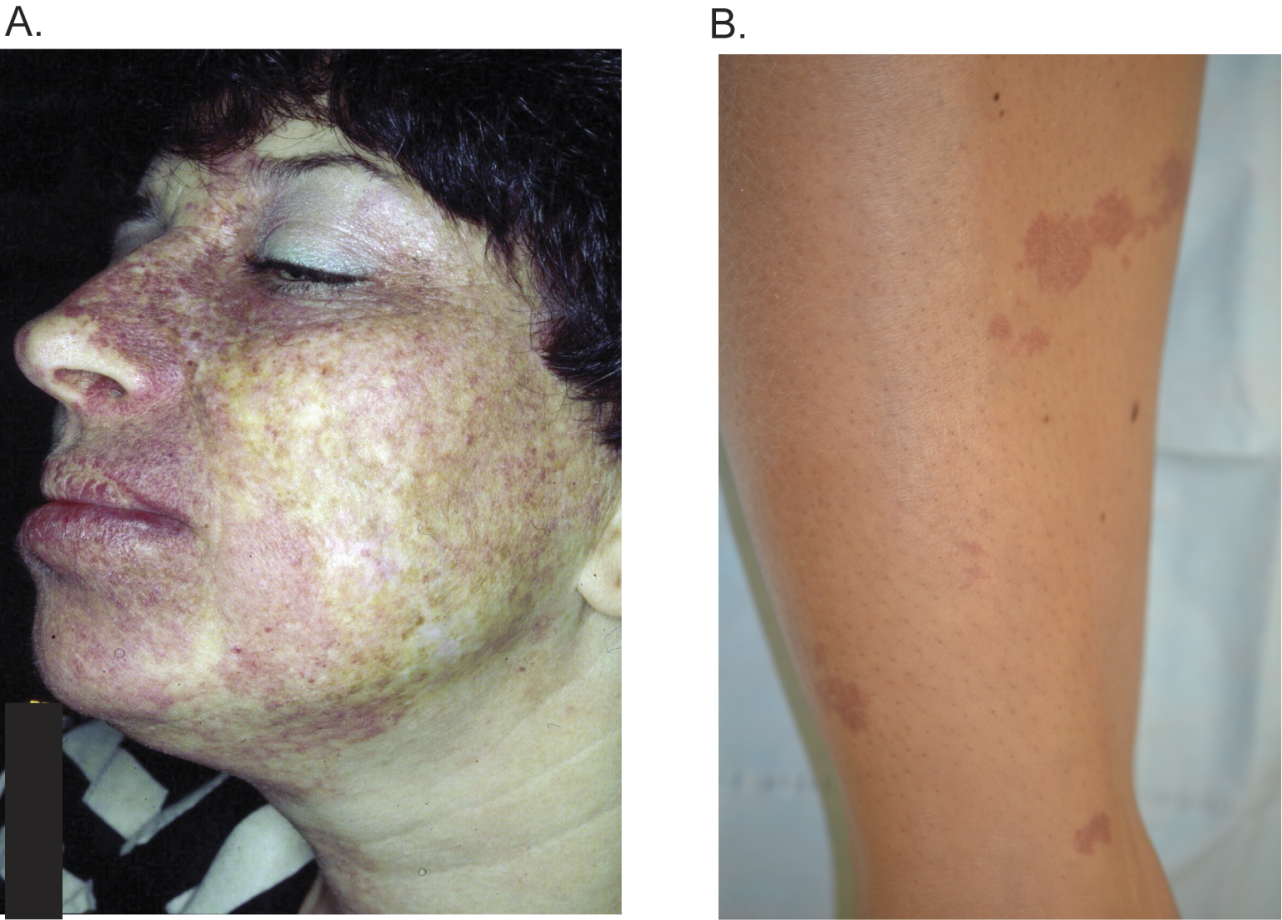
Case examples of port wine stains. Case #7 with a PWS of the facial V2 and V3 dermatomes (1a) harbored the novel variant *GNAQ* p.Arg183Gly. The PWS on the shin of case #9 (1b) was negative for a *GNAQ* or *RASA1* mutation.

A custom NGS panel was used to investigate the molecular genetics of PWS lesions from 12 individuals. All PWS cases were isolated, i.e. not associated with other known vascular anomalies or genetic syndromes (e.g. Sturge-Weber syndrome). PWS anatomical location and biopsy sites are described for each case in Tables 1 and 2. The facial PWS of case #7 is shown in Fig 1. Skin tissue was isolated from untreated PWS skin or unaffected skin (detailed locations in Tables 1 and 2). In addition, unaffected control DNA extracted from peripheral blood was used (Table 2, “Healthy Female”). Genetic analysis results from affected and unaffected skin tissue from the same individual are summarized in Tables 2 and 3. The average NGS coverage for PWS skin was 1203 reads (range of 447–2845 reads). The average coverage for control skin was 1667 reads (range of 416–2762 reads). Digital PCR was also used to determine the mutant allele frequency, and samples were considered positive if either the NGS or the dPCR data yielded a mutant allele frequency of >1%. Negative samples were <1% for both assays.

**Table 2:** Custom NGS and digital PCR results of the *GNAQ* somatic variant, *p*.*Arg183Gln*, *c*.*548G>A*.

Case		Biopsy Site	Custom Capture^1^	dPCR^2^	*GNAQ* ^3^
1	PWS	**L scalp**	**2.66**	**4.86 ± 0.60**	**Positive**
	Ctrl	R scalp	0.07	0.69 ± 0.97	
2	PWS	**L lower back**	**5.80**	**6.35 ± 0.07**	**Positive**
	Ctrl	R lower back	N/A	0.44 ± 0.10	
3	PWS	R wrist	0.07	0.18 ± 0.15	Negative
	Ctrl	L wrist	0.04	0.18 ± 0.01	
4	PWS	**L scalp**	**1.76**	**1.61 ± 0.37**	**Positive**
	Ctrl	R scalp	0.00	0.58 ± 0.41	
5	PWS	**R retroauricular**	**3.31**	**5.44 ± 0.92**	**Positive**
	Ctrl	L retroauricular	0.00	0.10 ± 0.04	
6	PWS	**L breast**	**3.98**	**3.69 ± 0.03**	**Positive**
	Ctrl	R breast	0.00	0.14 ± 0.01	
7	PWS	L scalp	0.00	0.00	other *GNAQ*
	Ctrl	R scalp	0.00	0.00	
8	PWS	**R buttock**	**2.99**	**6.15 ± 0.44**	**Positive**
	Ctrl	L buttock	N/A	0.49 ± 0.44	
9	PWS	R shin	0.13	0.11 ± 0.07	Negative
	Ctrl	L shin	N/A	0.00 ± 0.06	
10	PWS	**L scalp**	**1.73**	**0.85 ± 0.04**	**Positive** *
	Ctrl	R scalp	N/A	0.28 ± 0.03	
11	PWS	**L forearm**	**5.12**	**5.34 ± 0.20**	**Positive**
	Ctrl	R forearm	N/A	0.80 ± 1.20	
12	PWS	**R scalp**	**7.42**	**7.42 ± 0.04**	**Positive**
	Ctrl	L scalp	0	0.63 ± *	
**WildType**		Blood	0.06	0.22 ± 0.12	Negative

Abbreviations: R, right; L, left; dPCR, digital PCR; Ctrl, unaffected contralateral tissue

^1^the percentage for the number of variant containing reads over total reads

^2^mean% ± SD

^3^ *GNAQ* mutation interpretation.

For each participant, one intra-lesion biopsy was performed on PWS skin; a second biopsy was performed on apparently normal skin of the contralateral, corresponding body region.

* dPCR detection limit is 1%. However, we interpreted this case as positive by combining NGS data and dPCR value (close to 1%).

**Table 3:** Novel somatic variant found in *GNAQ*.

Location	Case	Tissue	Custom Capture (%)	dPCR (%)
*GNAQ* p.Arg183Gly, c.547C>G	*7*	PWS	**4.05**	**3.76 ± 0.37**
		Ctrl	0.00	0.18 ± 0.04
		healthy control	0.00	0.36 ± 0.35

Abbreviations: dPCR, digital polymerase chain reaction; Ctrl, unaffected tissue; N/A, no data available. Novel variants were not found in EVS (Exome Variant Server), COSMIC (Catalogue of Somatic mutations in Cancer) or ExAC (Exome Aggregation Consortium).

### Somatic *GNAQ* variants identified in most cases

**Fig 2:**
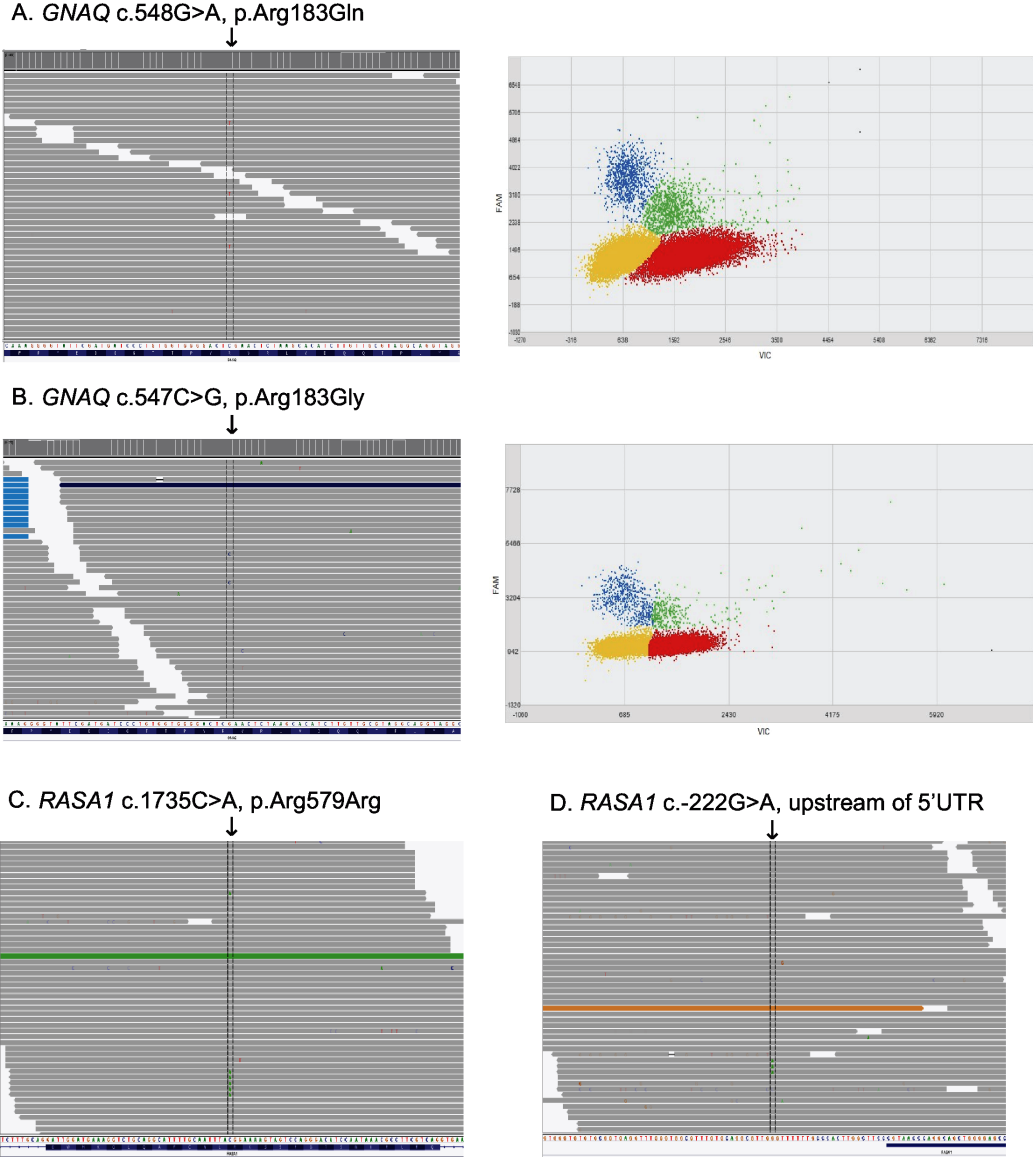
NGS and dPCR results for *GNAQ* and *RASA1* variants. For 2a and 2b, left panels depict the NGS data with the respective somatic *GNAQ* mutation (arrow). Right panels show corresponding digital PCR results analyzed using the QuantStudio 3D AnalysisSuite software. Relative intensities of FAM were plotted against VIC. The mutant allele (blue) depicts the level of somatic mosaicism in the sample versus the wild type allele (red), both alleles (green), and no amplified alleles (yellow). For 2c and 2d, NGS data with the respective somatic *RASA1* mutation (arrow) are shown from cases 5 and 7, respectively.

Of 12 PWS skin samples, 9 (75%) harbored the known *GNAQ* c.548G>A, p.Arg183Gln somatic change (Fig 2), with a mutation frequency of >1%. NGS captured data showed mutant allele frequencies between 1.73–7.42% in affected tissues, 0.00–0.13% in control skins, and 0.06% in healthy control DNA. Two cases had a *GNAQ* c.548G>A mutation frequency less than 0.2% by NGS indicating that they were negative for this mutation. Digital PCR results for the *GNAQ* c.548G>A, p.Arg183Gln variant were highly concordant with the NGS panel data, with mutant allele read frequencies ranging between 0.85% ± 0.04 and 7.42% ± 0.04 in affected tissues positive by NGS, 0.00% ± 0.06 and 0.80% ± 1.20 in control skins, and 0.22% ± 0.12 in healthy control DNA (Table 2). Two cases of isolated PWS of the limbs (right wrist from Case #3 and right shin from Case #9, Fig 1B) were negative for somatic *GNAQ* variants based on the 1% limit of detection for both assays (NGS and digital PCR).

Interestingly, case #7 was found to have a novel *GNAQ*, c.547C>G, p.Arg183Gly variant (Fig 2 and Table 3). This individual is a woman of Russian descent with a PWS involving the left V2 (maxillary trigeminal branch) and bilateral V3 (mandibular trigeminal branch) dermatomes. The novel *GNAQ* variant was present in 4.05% of reads from the custom NGS panel in the PWS tissue sample and was confirmed using digital PCR (3.76% ± 0.37). This novel variant is highly conserved and was predicted to be damaging by SIFT, PolyPhen, and Mutation Taster. The control tissue from this patient did not harbor the mutant allele nor did the healthy control (Table 3).

### Somatic *RASA1* variants identified

Two novel somatic *RASA1* variants were identified using the NGS panel in PWS samples from Case #5 and #7 (Fig 2). Case #5 harbored variant c.1735C>A, p.Arg579Arg, with a frequency of 1.8% in affected tissue and 0.0% in control tissue. Case #7 was found to harbor a somatic *RASA1* variant slightly upstream of the 5’UTR (c.-222G>A), with a 1.9% allele frequency in affected tissue and 0.0% in control tissue. Both variants were not predicted to have functional consequences on splicing (Human Splicing Finder [14]), translation or transcription factor binding according to prediction programs (NetStart 1.0 Prediction Server, CBS, Lyngby, Denmark). Therefore, further confirmation using digital PCR was not performed.

## Discussion

In most cases, PWS occur as sporadic, isolated lesions which appear to follow the embryonic vasculature of the face [15]. It has been proposed that lack of innervation in these vessels or multi-lineage developmental field defects might be responsible for the pathogenesis of PWS [1,16].

*GNAQ* was included in the custom NGS panel to identify the prevalence of a previously identified p.Arg183Gln variant in our cohort and to potentially identify novel *GNAQ* variants in PWS. Shirley *et al*. demonstrated that PWS are caused by a somatic activating mutation in *GNAQ* c.548G>A, p.Arg183Gln in 88% of samples (23 of 26) from patients with Sturge-Weber syndrome and 92% of samples (12 of 13) from individuals with PWS alone [5]. Also Nakashima et al. detected the presence the same mutation in 80% (12 of 15) of brain tissue from indivisuals with Sturge-Weber Syndrome (SWS) [17]. In our study, the somatic *GNAQ* p.Arg183Gln mutation was identified in 75% of PWS tissue samples (9 of 12 individuals). While the frequency of the mutant allele in affected skin tissue was 1.0–18.1% in Shirley et al’s cohort, it was 1.0–7.42% in our cohort. Given the use of different technologies in these cohorts, the results are similar, but the ratio is lower in our group. The difference could be due to the inherent mixed cellular composition of the tissues studied. If the dermal layers were further isolated from the subcutaneous fat and epidermis, the frequency of the mutant allele would likely have been higher. Finally, we used a subtractive correction method to eliminate control variants as well as background noise from the NGS panel results [12,13]. Importantly, dPCR confirmed the low level mosaic variants identified by the NGS panel.

Interestingly, we also found that 1 of 12 cases (8.3%) carried a novel *GNAQ* variant (c.547C>G, p.Arg183Gly) in the PWS skin (Case #7). This novel variant, p.Arg183Gly, has not been previously reported in any databases including COSMIC, and was predicted to be damaging by several prediction algorithms. This novel variation affects the same amino acid residue of the GNAQ protein described previously in PWS, arginine 183 [5]. This conserved residue is located in the guanosine triphosphate (GTP) binding pocket of all human Gα subunits. Here, the positive charge of arginine 183 plays a critical role in the hydrolysis of GTP by stabilizing the negative charge of the penta-coordinate phosphate intermediate, facilitating hydrolysis of the phosphate group and inactivating the protein [18]. Substitution of this residue to either a polar glutamine or hydrophobic glycine residue likely impairs hydrolysis such that GNAQ remains in its active GTP-bound form, resulting in the observed PWS phenotype. Other substitutions resulting in anything other than arginine at this residue would likely result in a PWS phenotype. Although this patient is of Russian decent, the mutation is a post-zygotic somatic mutation and its prevalence should not be influenced by ethnic group. Because most current GNAQ digital PCR assays screen for the p.Arg183Gln, this novel mutation would likely be missed. The prevalence of this particular somatic mutation in PWS tissue may be higher as molecular techniques become more inclusive (ie. NGS).

At a molecular level, significant ERK (Extracellular Signal-Regulated Kinases) activation has been observed in human embryonic kidney cells transfected with *GNAQ* p.Arg183Gln, as compared with cells transfected with non-mutant *GNAQ* [5]. The mutation did not seem to activate other MAPK (Mitogen-Activated Protein Kinase) pathway members, as well as the AKT (Protein Kinase B) signaling pathway. More recent data report the activation of ERK and JNK (c-Jun N-terminal kinase) in 19 of 19 PWS samples. JNK activation levels appeared correlated to the progressive development of PWS [19]. It is likely that the novel *GNAQ* p.Arg183Gly variant is also an activating mutation because it disrupts the same critical arginine residue responsible for facilitating GTP hydrolysis and inactivation of *GNAQ*.

The role of *GNAQ* gain-of-function in causing the PWS phenotype has not yet been unraveled. Knowing which cell lines harbor the mutation would help guide hypotheses on possible molecular mechanisms at the origin of this disease.

Among the cases positive for *GNAQ* mutations, we found no correlation between the anatomical site and the mutation level. Both cases with a small PWS of the limbs were negative for *GNAQ* and *RASA1* mutations. The mixed cellular population may have led to the inability to detect cells with *GNAQ* mutations in these two individuals. Lian *et al*. recently described novel somatic mutations in the genes *SMARCA4*, *EPHA3*, *MYB*, *PDGFR-β*, and *PIK3CA*, in addition to the known *GNAQ* variant, found in a case of isolated PWS by exome sequencing [20]. These genes are involved in embryonic venous specification, angiogenesis, and proliferation of vascular smooth muscle cells [18]. The inclusion of these genes in future NGS panels may help identify additional genetic causes in PWS cases who do not have a somatic *GNAQ* variant.

This study also identified two novel somatic *RASA1* variants (p.Arg579Arg and c.-222G>A, in case #5 and #7 respectively). Both variants should not have functional consequences on splicing, translation or transcription factor binding according to prediction programs. These PWS cases involved cervicofacial dermatomes and also harbored *GNAQ* somatic variants. No additional *RASA1* germline mutations were found in these two participants. To our knowledge, these lesions are isolated and not associated with high-flow lesions. No data suggest that *RASA1* changes are the main genetic determinants in the pathogenesis of the studied PWS cohort; interestingly, recently published studies also report that *RASA1* does not seem to be involved as a genetic determinant of most familial PWS [3]. This helps confirm that individuals with isolated PWS do not need *RASA1* testing and phenotypes between the two are different.

This study identified novel mutations in both genes within our PWS cohort. Discovering a novel *GNAQ* variant, as well as documenting 9 of 12 cases harboring the known *GNAQ* mutation (p.Arg183Gln), corroborates the hypothesis that *GNAQ* mutations represent one of the most important PWS pathogenic factors in the early stages of development. However, *GNAQ* mutations were not identified in all cases, suggesting that other genetic factors are also involved in the pathogenesis of PWS.
